# The prognostic value and oncogenic functions of WDR12 and its validation in osteosarcoma

**DOI:** 10.1186/s12935-026-04291-6

**Published:** 2026-04-08

**Authors:** Zhiming Zhang, Ruiqi Chen, Zhongyue Liu, Binfeng Liu, Lin Mei, Chi Yin, Lu Wan, Zhihong Li

**Affiliations:** 1 Trauma Center, The Second Xiangya Hospital of Central South University, Changsha, 410011 Hunan China; 2 Hunan Key Laboratory of Tumor Models and Individualized Medicine of The Second Xiangya Hospital of Central South University, Changsha, 410011 Hunan China; 3 Hunan Engineering Research Center of AI Medical Equipment, The Second Xiangya Hospital of Central South University, Changsha, 410011 Hunan China; 4 Department of Neurosurgery, The Second Xiangya Hospital of Central South University, Changsha, 410011 Hunan China; 5 Trauma Center, The Second Xiangya Hospital of Central South University, Changsha, 410011 Hunan China

**Keywords:** Osteosarcoma, Bone tumor, WDR12, Prognosis, Immune

## Abstract

Osteosarcoma (OS) stands as the predominant malignant primary bone tumor. WDR12 plays a role in diverse biological processes. Nonetheless, the specific function of WDR12 in OS has not been clearly elucidated. Herein, we found that WDR12 is overexpressed and closely associated with poor prognosis of OS. A diagnostic nomogram based on WDR12 and clinical features of OS could effectively predict the overall survival rate of OS patients. Through functional enrichment analysis, multiple potential pathways related to WDR12 expression in tumorigenesis have been identified. Additionally, the expression level of WDR12 is correlated with the tumor immune microenvironment, immune therapy response, and drug treatment response in OS. Importantly, in vitro experiments demonstrated that downregulation of WDR12 significantly inhibited the proliferation and migration abilities of OS cells and in vivo experiments also confirmed that overexpression of WDR12 could promote tumor growth. Hence, WDR12 has the capacity to emerge as a novel prognostic biomarker and therapeutic target for OS.

**Supplementary Information:**

The online version contains supplementary material available at 10.1186/s12935-026-04291-6.

## Introduction

Osteosarcoma (OS) is a malignant tumor that arises from mesenchymal cells, primarily afflicting adolescents and children [1]. The most common sites for this tumor are the metaphysis of long bones, distal femur, and proximal tibia. Initial symptoms of OS are often subtle, mainly presenting as localized pain and swelling [2]. Over time, the pain becomes constant and severe, particularly exacerbated at night. OS is an aggressive cancer that spreads quickly and has a tendency to metastasize. Approximately 20% of patients are found to have distant metastases at the time of diagnosis [3]. The current treatment options for OS include surgery, radiotherapy, and chemotherapy [4]. The improvements in medical technology result in better 5-year survival rates, while significant progress in combating this disease remains challenging. Key obstacles that persist include the limitations of limb-salvage surgery, preserving limb function, managing chemotherapy-related adverse reactions and drug resistance, addressing low sensitivity to radiotherapy and radiation-induced damage, uncertainty surrounding immunotherapy, and effectively managing OS-related pain [5]. Therefore, identifying reliable prognostic biomarkers and novel therapeutic targets is critical for improving clinical outcomes in OS.

With the rapid development of high-throughput sequencing and bioinformatics approaches, increasing attention has been focused on elucidating the molecular mechanisms underlying tumor initiation and progression and on identifying key regulatory genes involved in cancer development [6]. Among these, genes regulating fundamental cellular processes such as ribosome biogenesis and cell proliferation have emerged as important contributors to tumorigenesis.

WDR12, a member of the WD repeat protein family, exhibits extensive-expression throughout embryonic development and demonstrates prominent expression in the thymus and testis tissues of adult mice [7]. In mammals, WDR12 plays a critical role in ribosome biogenesis and cell proliferation. It forms a stable complex, known as PeBoW, with Pes1 and Bop1 and actively contributes to the maturation process of ribosomal 28 S rRNA, 5.8 S rRNA, and the 60 S large subunit [8]. WDR12 plays a significant role in regulating normal cell proliferation and survival, and its involvement in various malignant tumors has been progressively uncovered. For instance, Jun-Liang Li et al. demonstrated that elevated expression of WDR12 is linked to shorter overall survival and lower disease-free survival in glioblastoma patients, and the knockout of WDR12 promotes apoptosis and proliferation of glioblastoma cells [9]. Meanwhile, Yancun Yin et al. revealed that WDR12 can maintain the proliferation and migration of Hepatocellular carcinoma cells by promoting the Akt-mTOR-S6K1 signaling pathway [10]. However, despite accumulating evidence supporting the oncogenic role of WDR12 in multiple malignancies, its biological function, clinical relevance, and potential therapeutic implications in OS remain largely unexplored. In particular, it is unclear whether WDR12 expression is associated with OS prognosis, tumor progression–related pathways, immune microenvironment characteristics, or therapeutic response, representing a critical gap in current knowledge.

In this study, we hypothesized that WDR12 plays a tumor-promoting role in OS and may serve as a prognostic biomarker and potential therapeutic target. To test this hypothesis, we performed a comprehensive and integrative analysis combining public datasets and experimental validation. Specifically, we systematically evaluated WDR12 expression patterns and prognostic significance in OS using multiple independent cohorts, explored its potential molecular mechanisms through co-expression and functional enrichment analyses, and investigated its association with the tumor immune microenvironment, immunotherapy response, and chemotherapeutic drug sensitivity. Furthermore, in vitro and in vivo experiments were conducted to validate the functional role of WDR12 in OS progression. Collectively, this study provides novel insights into the biological and clinical significance of WDR12 in OS.

## Materials and methods

### Data acquisition and preprocessing

The survival data, clinical characteristics, and gene expression profiles of OS patients were obtained from the Therapeutically Applicable Research to Generate Effective Treatments (TARGET) database (https://ocg.cancer.gov/programs/target). Publicly available GEO datasets (GSE16088, GSE19276, and GSE28424) were retrieved from the Gene Expression Omnibus (GEO, https://www.ncbi.nlm.nih.gov/geo/) to evaluate WDR12 expression in OS tissues and cell lines.

Specifically, GSE16088 includes gene expression profiles from 14 human OS tumor samples and 6 normal bone samples [11]; GSE19276 consists of 44 OS samples and 5 normal tissue samples [12]; and GSE28424 contains 19 OS cell lines and 4 normal bone cell lines used as controls [13]. These datasets were selected because they contain both OS and corresponding normal controls and have been widely used in previous OS transcriptomic studies.

For prognostic validation, the GSE21257 dataset (53 OS patients with survival information) was obtained from GEO, and the TCGA sarcoma (TCGA-SARC) dataset, including gene expression and survival data from 255 sarcoma patients, was downloaded from The Cancer Genome Atlas (TCGA, https://portal.gdc.cancer.gov/) [14]. Due to the lack of an OS-specific cohort in TCGA, TCGA-SARC was used as an external validation dataset, as reported in multiple previous OS-related studies. Detailed clinical characteristics of patients from all datasets are summarized in Tables S1–S6.

### Data integration, normalization, and batch-effect correction

For RNA-seq data, Fragments Per Kilobase per Million mapped reads (FPKM) values were transformed into Transcripts Per Million (TPM) to improve comparability across samples. When integrating gene expression data from different cohorts or platforms, normalization and batch-effect correction were performed prior to downstream analyses. Specifically, expression matrices were log2-transformed [log2(TPM + 1)], and batch effects were adjusted using the ComBat algorithm implemented in the “sva” R package, when applicable. Microarray datasets from GEO were analyzed independently to avoid cross-platform bias and were not directly merged with RNA-seq datasets. Pan-cancer expression analyses comparing tumor tissues from TCGA with normal tissues from GTEx were conducted using the Xiantao Academic Online Tools (https://www.xiantao.love/products), which provide uniformly processed and batch-corrected TCGA–GTEx expression data based on standardized pipelines. When comparing WDR12 expression across 33 cancer types, multiple comparisons were adjusted using the Benjamini–Hochberg false discovery rate (FDR) method. An adjusted P value (FDR) < 0.05 was considered statistically significant. All analyses were performed on normalized expression values to minimize technical variability.

### Prognostic analysis of WDR12 in OS

The predictive significance of WDR12 for OS survival was assessed through the log-rank test and Cox regression model, utilizing data from the TARGET, GSE21257, and TCGA databases. Univariate Cox analysis and multivariate Cox analyses were employed to detect whether the expression of WDR12 and other parameters could be considered independent prognostic factors for OS.

### Nomogram construction

Nomograms are widely utilized in the prognosis prediction of cancer. In order to predict the prognosis of OS patients more accurately, we employed the R package “rms” to develop a novel nomogram that could predict the 1-year, 3-year, and 5-year overall survival rates for OS. Moreover, the R packages “caret,” “timeROC,” and “rmda” were further used to draw calibration charts, ROC curves and decision curve analysis (DCA), respectively, to evaluate the prediction effect of the nomogram.

### Co-expression analyses and functional enrichment analyses

Studying co-expressed genes is an effective method to further analyze the function and molecular mechanism of specific genes. Correlation analysis was performed using the Pearson correlation coefficient method to explore co-expressed genes that were positively and negatively correlated with WDR12. The strength of the correlation between genes is represented by the correlation coefficient. According to the correlation coefficient, the top 5 positively correlated co-expressed genes and the top 5 negatively correlated co-expressed genes are selected and visualized using a circus diagram. To further explore the potential mechanism of WDR12 in OS, a functional enrichment analysis was conducted. Based on co-expressed genes, Gene Ontology (GO) and Kyoto Encyclopedia of Genes and Genomes (KEGG) [15] enrichment analysis was performed using The Xiantao Academic Online Tools. Additionally, the Gene set enrichment analysis (GSEA) in high and low WDR12 expression groups was conducted by the R package “clusterProfiler” with the reference dataset from the MSigDB. Multiple hypothesis testing was corrected using the Benjamini–Hochberg method, and pathways with an adjusted P value (FDR) < 0.05 were considered significantly enriched.

### Immunological analysis

A variety of analysis methods were employed to explore the relationship between WDR12 expression and the tumor microenvironment in OS. The “estimateScore” algorithm was utilized to calculate the stromal score, immune score, ESTIMATE score and tumor purity of all OS samples. Also, the values and correlations of these scores among different WDR12 expression groups were compared. The CIBERSORT algorithm was used to determine the composition and quantification of immune cells in the tumor microenvironment of OS and to compare the proportions of immune cells between high and low WDR12 expression groups. The R package “GSVA” was used to perform single-sample GSEA (ssGSEA) to compare immune cell infiltration between different WDR12 expression groups. In addition, this study also explored the connection between WDR12 expression levels and immune checkpoint genes.

### Comparison of treatment response under different expression levels of WDR12

To help evaluate immunotherapy response in OS patients, we calculated the Tumor Immune Dysfunction and Exclusion (TIDE) score, T cell dysfunction score, T cell exclusion score, and microsatellite instability (MSI) score based on the TIDE website (http://tide.dfci.harvard.edu/). The Genomics of Drug Sensitivity in Cancer (GDSC) database is a comprehensive resource that integrates large-scale drug sensitivity data with genomic and transcriptomic information from a wide array of cancer cell lines. It enables the identification of molecular markers of drug response and helps predict therapeutic efficacy in cancer patients. The “pRRophetic” algorithm leverages this database to estimate drug sensitivity by correlating gene expression profiles from patient tumor samples with drug response data from cell lines in the GDSC dataset. This approach has been widely used in cancer research to predict IC50 values (a measure of drug potency) for various therapeutic agents based on tumor transcriptomic data. In this study, the “pRRophetic” software package was used to predict the half-maximal inhibitory concentration (IC50) value of a drug based on gene expression profiles obtained from GDSC and then compare the therapeutic response to different drugs in OS patients with high- and low- WDR12 expression.

### Transcriptome analysis at the single-cell level

The single-cell RNA sequencing datasets GSE152048 and GSE162454 were analyzed using the Seurat software package following the standard pipeline [16, 17]. Subsequently, gene expression was normalized using LogNormalize with a scale factor of 10,000. The identification of 2000 highly variable genes was performed using the FindVariableGenes function. Cell subgroups were annotated using the annotation method described in a previous publication [18].

### Tissue collection and protein extraction

Tumor and adjacent normal tissue samples were obtained from surgically resected or biopsied OS patients and were confirmed by pathological examination. This study was conducted in accordance with the Declaration of Helsinki and was approved by the Ethics Committees of The Second Xiangya Hospital, China (Approval No.2023-Z0477). Written informed consent was obtained from all participants prior to sample collection. Proteins were extracted from tissue specimens using RIPA lysis buffer (P0013B, Beyotime) supplemented with protease and phosphatase inhibitors. The protein samples were denatured at 100 °C and subsequently subjected to Western blot analysis.

### Cell lines and cell culture

As described in previous articles, the human normal osteoblast cell line (Human fetal osteoblasts, hFOB1.19), OS cell lines (143B, U2OS, and HOS cell lines) and HEK-293T cell lines were purchased from the American Type Culture Collection (ATCC) and cultured in appropriate media according to ATCC instructions [19]. The hFOB1.19 cells were cultured at 34 °C, while the rest of cell lines were cultured at 37 °C.

### RT−qPCR

Total RNA extraction and RT-qPCR were performed as our previous study described [20]. The primer sequences are provided in Table S7. All data were normalized to the expression of the housekeeping gene GAPDH.

### Western blot

After protein extraction, proteins in cell lysates were separated by sodium dodecyl sulfate-polyacrylamide gel electrophoresis (SDS-PAGE) and then transferred to the PVDF membrane. Subsequently, the membranes were blocked with 5% skim milk powder for 1 h at room temperature and then incubated with primary antibodies. The primary antibodies used for western blot were WDR12 (ab111955) from Abcam and GAPDH (60004-1-Ig) from Pronteintech. After incubation with peroxidase-conjugated secondary antibodies, HRP activity was detected using an ECL detection system (Bio-Rad, USA).

### Lentiviral infection

The sequences of shRNA targeting WDR12 were as follows: WDR12-shRNA#1: 5′-CCTTGGAGAAAGTCAATAA-3′ and WDR12- shRNA#2: 5′-GCTGATTTCAGGATCTTTA-3′. The shRNA plasmid was co-transfected into HEK-293T cells along with pSPAX2 and pMD2G packaging materials. The lentivirus-containing supernatant was collected at 48- and 72 h post-transfection and stored at −80 °C. The lentivirus was filtered and used to infect the target cells overnight. Subsequently, the cells were screened with a fresh culture medium containing 2 µg/mL puromycin. Finally, the knockdown efficiency was determined by Western blot.

### MTT

OS cells were seeded at a density of 1,000 cells per well in a 96-well plate. The plate was then placed in a cell culture incubator at 37 °C with 5% CO2. After a specified period of culturing, the old culture medium was discarded, and each well was supplemented with 10 µl of MTT and 90 µl of the medium. The plate was incubated for four hours in the cell culture incubator. Next, the supernatant was removed, and each well was treated with 100 µl of DMSO. Finally, the plate was gently shaken, and the absorbance (OD) value of each well was measured using a microplate reader.

### Clone formation

OS cells were seeded at a density of 1 × 10^3^ cells per well in 6-well plates and cultured using a complete medium for a duration of 7–10 days. Afterward, the cells were washed with PBS, fixed with 4% formaldehyde for 10 min, and subsequently stained with crystal violet for 30 min. Finally, cell counting was performed using ImageJ.

### Wound healing assay

OS cells were seeded in 6-well plates. Once the cells reached 90% confluence, scratches were made in the cell monolayer using a sterile 200 µl pipette tip. Subsequently, the cells were washed with PBS to remove any detached cells, and the culture medium was replaced with FBS-free DMEM. The area of each scratch was then photographed at specific time points and measured using ImageJ.

### Transwell

The migratory and invasive capabilities of OS cells were evaluated using transwell chambers with an 8 μm pore size in a 24-well plate. A total of 1 × 10^4^ 143B cells or HOS cells were seeded in 200 µl of DMEM in the upper compartment of the transwell chamber, while 600 µl of DMEM containing 10% FBS was added to the lower compartment. For invasion assays, matrix gel was pre-applied to the upper compartment of the transwell chamber prior to cell seeding. Following a 48-hour incubation in a cell culture incubator, the cells remaining in the upper compartment were removed, and the cells that had migrated through the filter were fixed using 4% paraformaldehyde. Then, the migrated cells on the filter were stained with crystal violet solution, photographed under a microscope, and quantified using ImageJ.

### Animal study

All animal experiments were performed in accordance with institutional guidelines for animal care and use and were approved by the Institutional Animal Care and Use Committee (IACUC) of The Second Xiangya Hospital, China (Approval No. 20230301). Five-week-old male BALB/c nude mice were used for the xenograft experiments. A total of 1 × 10^6 wild-type 143B cells or WDR12 knockdown cells were suspended in DMEM medium and subcutaneously injected into the dorsal flanks of the nude mice. After 3 weeks, the mice were humanely euthanized by carbon dioxide inhalation followed by cervical dislocation as a secondary method, in accordance with approved protocols. Tumor tissues were then excised and collected for measurement of tumor weight and volume.

### Statistical analysis

All statistical analyses were conducted using R software (version 4.1.1) and GraphPad Prism (version 8.0.0). All in vitro functional assays, including cell proliferation, migration, and invasion assays, were performed with at least three independent biological replicates. Each experiment was repeated independently to ensure reproducibility, and representative results are shown. Kaplan–Meier survival curves were generated, and differences were evaluated using the log-rank test. Univariate and multivariate Cox proportional hazards regression analyses were performed to estimate hazard ratios (HRs) and 95% confidence intervals (CIs). In multivariate models, available clinical covariates (age and sex) were included to evaluate the independent prognostic value of WDR12. T-tests or Wilcoxon tests were employed to compare two independent groups, while the Kruskal-Wallis test and one-way ANOVA tests were used to analyze differences among multiple groups. A two-sided P value < 0.05 was considered statistically significant.

## Results

### WDR12 gene expression analysis

**Fig. 1:**
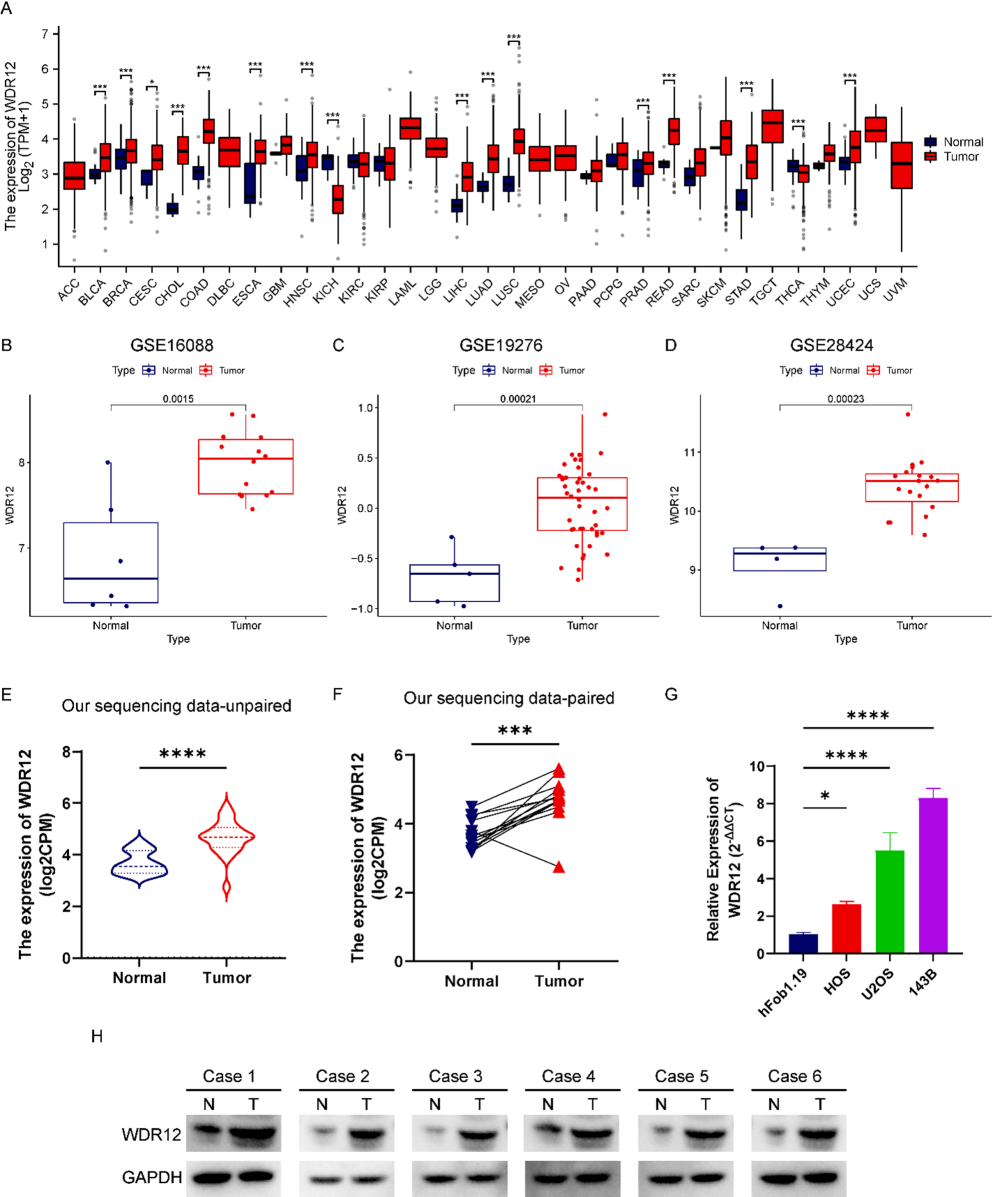
The expression of WDR12 is upregulated in osteosarcoma. (**A**) WDR12 mRNA expression in malignant tumor and normal tissues based on the TCGA database. (**B**) WDR12 mRNA expression in normal and OS tissues based on GSE16088. (**C**) WDR12 mRNA expression in normal and OS tissues based on GSE19276. (**D**) WDR12 mRNA expression in normal and OS tissues based on GSE28424. (**E-F**) WDR12 expression in normal tissue and OS tissue based on transcriptome sequencing. (**G**) WDR12 mRNA expression in OS cell lines (143B, U2OS, and HOS) compared with the normal cell line hFob1.19 by RT−qPCR. (**H**) The protein expression of WDR12 in six paired osteosarcoma (T: tumor) and normal tissues (N: normal) were lysed and analyzed by Western blotting

Initially, we investigated the expression levels of WDR12 in various cancer types, revealing distinct patterns across malignancies (Fig. 1A). The results showed that WDR12 exhibits high expression in BLCA, BRCA, CESC, CHOL, COAD, ESCA, HNSC, LIHC, LUAD, LUSC, PRAD, READ, STAD, and UCEC while exhibiting low expression in KICH and THCA (Fig. 1A). Next, we assessed the expression of WDR12 in OS using the GSE16088, GSE19276, and GSE28424 datasets. The results revealed a significant upregulation of WDR12 in OS tissues compared to normal tissues (Fig. 1B-D). Additionally, our tissue sequencing data further confirmed the increased expression of WDR12 in OS, whether in unpaired or paired tissue samples (Fig. 1E-F). Similarly, RT-qPCR analysis showed WDR12 overexpression in OS cell lines (143B, U2OS, and HOS) compared to the normal cell line (hFob 1.19) (Fig. 1G). Consistently, protein expression of WDR12 was significantly elevated in OS tissues compared to adjacent normal tissues (Fig. 1H). Collectively, these findings underscore the upregulation of WDR12 in OS.

### Prognostic potential of WDR12 in OS

**Fig. 2:**
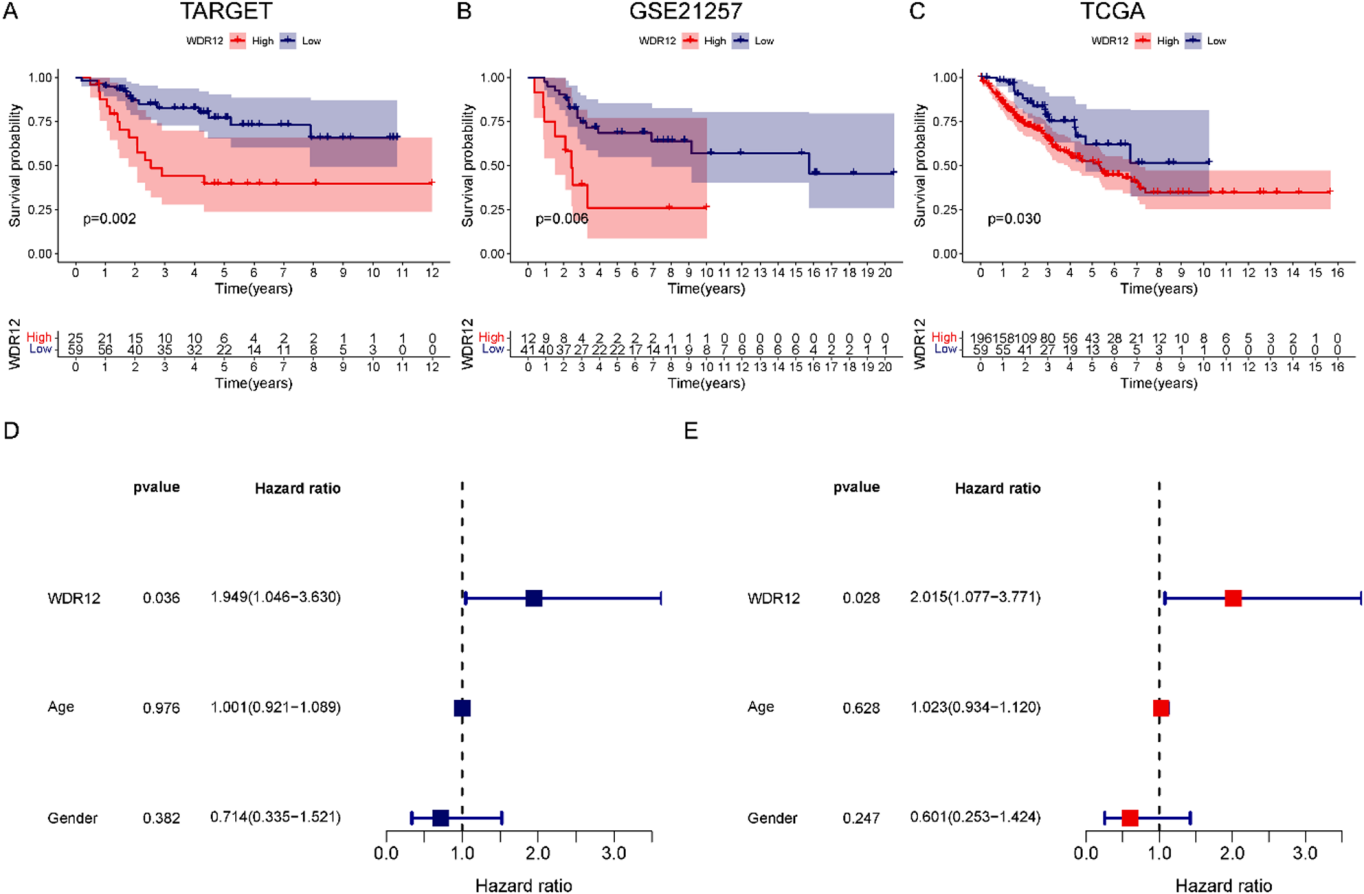
Prognostic analysis of WDR12 expression in OS. (**A**) KM curves of over-survival for OS patients with different WDR12 expressions based on the TARGET database. (**B**) KM curves of over-survival for OS patients with different WDR12 expressions based on the GSE21257 database. (**C**) KM curves of over-survival for OS patients with different WDR12 expressions based on the TCGA database. TCGA-SARC was used as a supplementary validation cohort due to limited availability of large OS-specific TCGA datasets. (**D**) Univariate Cox regression analysis of WDR12 in the TARGET database. (**E**) Multivariate Cox regression analysis of WDR12 in the TARGET database

Then, we conducted a comprehensive analysis of the prognostic implications of WDR12 in OS. Using the TARGET database, which includes well-annotated OS clinical data, we observed that patients with low WDR12 expression exhibited significantly better overall survival compared with those with high WDR12 expression (*P* < 0.05, Fig. 2A). These findings were further validated in the independent GSE21257 cohort, which consists exclusively of OS patients, and consistently demonstrated that elevated WDR12 expression was associated with poorer prognosis (*P* < 0.05, Fig. 2B). In addition, a supportive prognostic analysis was performed using the TCGA-SARC dataset, which comprises multiple sarcoma subtypes, and a similar adverse prognostic trend associated with high WDR12 expression was observed (*P* < 0.05, Fig. 2C). Subsequently, univariate and multivariate Cox regression analyses based on OS-specific cohorts provided further evidence that WDR12 expression is an independent predictor of prognosis in OS (both HRs >1 and *P* < 0.05, Fig. 2D, E). Collectively, these results indicate that elevated WDR12 expression is associated with unfavorable clinical outcomes in OS and may serve as an independent prognostic indicator for OS patients.

### Construction of a nomogram

**Fig. 3:**
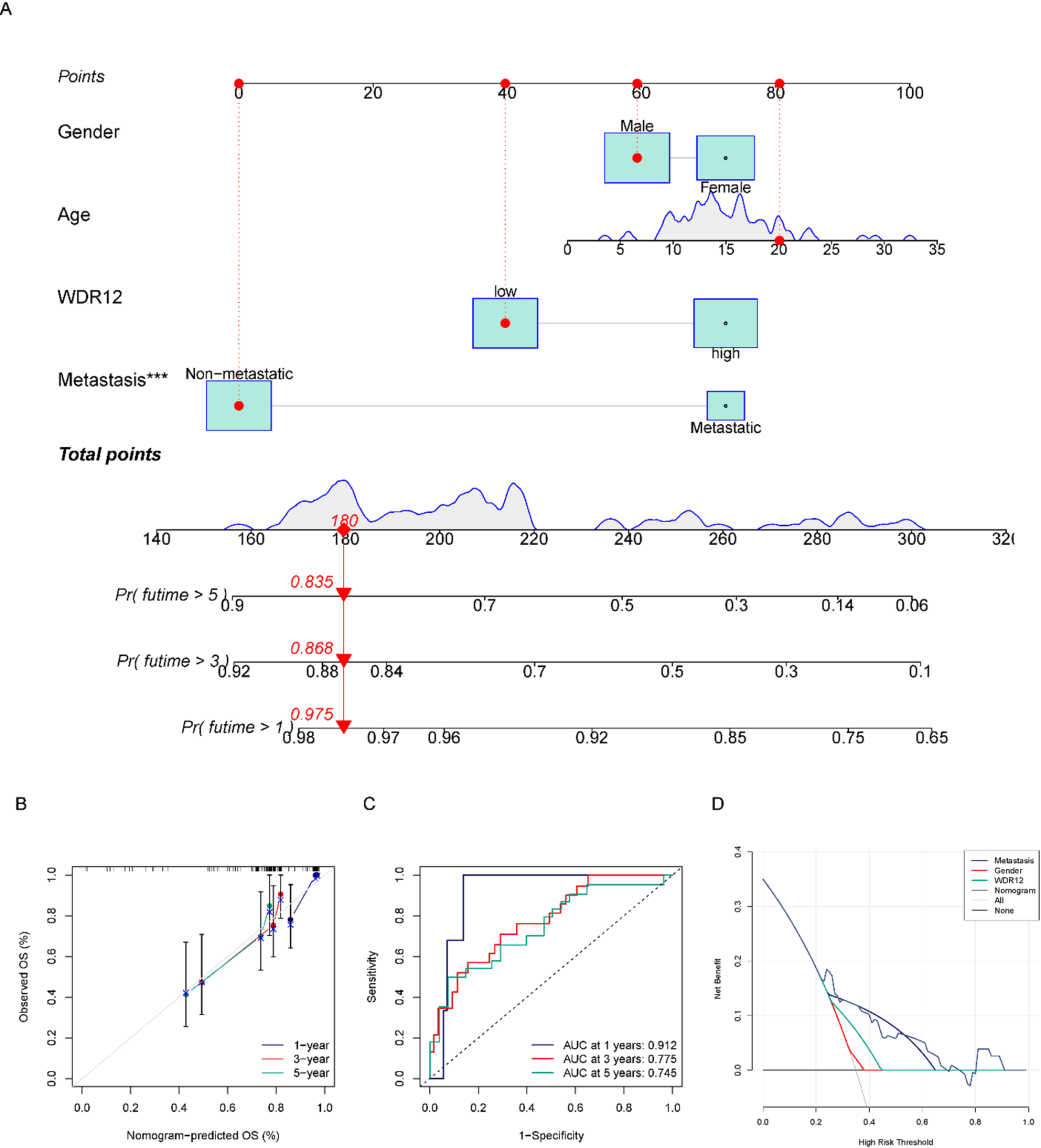
Prognostic prediction nomogram of WDR12. (**A**) A nomogram based on WDR12 expression level and independent clinical factors for overall survival prediction of OS. (**B**) Calibration curve. (**C**) The 1-, 3-, and 5-year AUC of the ROC curve of the nomogram. (**D**) Decision curve analysis plot

To enhance the accuracy of prognostic assessments for OS patients, we developed a novel nomogram that incorporates WDR12 expression levels and key clinical characteristics. The nomogram was constructed using patient age, gender, metastasis status, and WDR12 expression level, providing a predictive tool for estimating 1-year, 3-year, and 5-year survival rates of OS patients (Fig. 3A). To further evaluate the predictive ability of the nomogram, we additionally plotted the calibration curve, ROC curve, and DCA curve. The results showed that the calibration curve demonstrated a high level of agreement between the predicted survival rates from the nomogram and the actual survival rates of OS patients (Fig. 3B). The ROC curve indicated that the area under the curve (AUC) for the nomogram at 1 year, 3 years, and 5 years was 0.912, 0.775, and 0.745, respectively (Fig. 3C). Moreover, the DCA curve suggested that the nomogram significantly contributes to clinical decision-making (Fig. 3D). These findings highlight the robustness of the nomogram we developed.

### WDR12 co-expression analysis and functional enrichment analysis

**Fig. 4:**
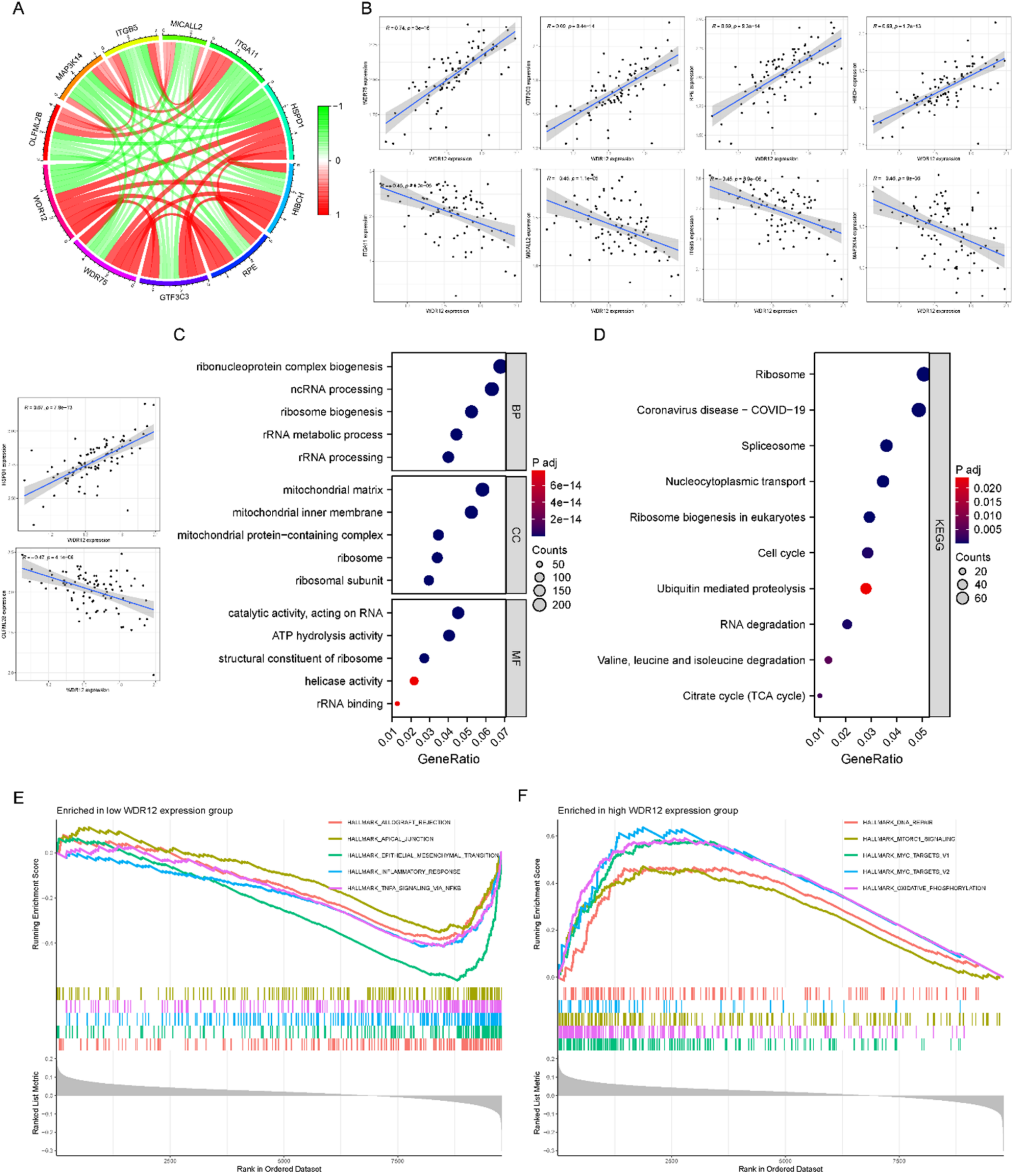
Functional enrichment analysis of WDR12 in OS. (**A**) The circle plot shows 5 positive and 5 negative genes that are most significantly co-expressed with WDR12 in OS. (**B**) The scatter plot of co-expressed genes associated with WDR12. (**C**) GO analysis. (**D**) KEGG analysis. (**E**) GSEA plot for the low WDR12 expression group: The upper panel shows running enrichment score (RES) curves for 5 significantly enriched hallmark pathways (allograft rejection, apical junction, epithelial-mesenchymal transition, inflammatory response, TNFα signaling via NFκB; curve colors match pathway labels); the middle panel displays the position of genes corresponding to each pathway in the WDR12-expression-ordered dataset (colored bars align with pathway curves); the lower panel represents the rank metric (reflecting the correlation direction between genes and WDR12 expression).(**F**) GSEA plot for the high WDR12 expression group: The upper panel shows RES curves for 5 significantly enriched hallmark pathways (DNA repair, MTORC1 signaling, MYC targets V1, MYC targets V2, oxidative phosphorylation; curve colors match pathway labels); the middle panel displays the position of genes corresponding to each pathway in the WDR12-expression-ordered dataset (colored bars align with pathway curves); the lower panel represents the rank metric (reflecting the correlation direction between genes and WDR12 expression)

The correlation between WDR12 and various genes was explored using Pearson correlation analysis. The findings indicated that WDR75, GTF3C3, RPE, HIBCH, and HSPD1 had a positive correlation with WDR12, while ITGA11, MICALL2, ITGB5, MAP3K14, and OLFML2B showed a negative correlation with WDR12 (Fig. 4A). Figure 4B illustrates the correlation of these genes with WDR12. Survival analysis further revealed the potential of these genes to serve as co-targets. The results showed that the expression levels of WDR75, GTF3C3, RPE, and HSPD1 were associated with poor prognosis in OS patients, while the expression of ITGA11, MICALL2, ITGB5, MAP3K14, and OLFML2B correlated with improved survival prognosis (Figure S1). To further validate the correlation analysis, we conducted PCR experiments to assess the expression levels of the co-expressed genes in 143B cells following shRNA-mediated silencing of WDR12. Consistent with our previous analysis, we observed that the expression levels of WDR75, GTF3C3, RPE, HIBCH, and HSPD1 were downregulated upon WDR12 knockdown, while the expression of ITGA11, MICALL2, ITGB5, MAP3K14, and OLFML2B was upregulated (Figure S1). Based on these co-expression relationships, GO, and KEGG enrichment analyses were conducted, and the respective results are presented in Fig. 4C-D. Additionally, we categorized individuals into high and low WDR12 groups based on the median expression level of WDR12 and conducted a GSEA analysis. The results of GSEA revealed that allograft rejection, apical junction, epithelial-mesenchymal transition, inflammatory response, and TNFα signaling via NFκB were significantly enriched in the low-WDR12 group (Fig. 4E). Conversely, the pathways such as DNA repair, MTORC1 signaling, MYC target V1, MYC target V2, and oxidative phosphorylation signaling pathway were significantly enriched in the high-WDR12 group (Fig. 4F). To further validate these findings, we selected representative genes from these pathways and assessed their expression levels following WDR12 silencing in 143B cells. Specifically, we examined the expression of RAD51 (DNA repair), MYC (MYC targets), mTOR (MTORC1 signaling), and ATP5F1 (oxidative phosphorylation). Consistent with our GSEA results, the expression levels of RAD51, MYC, and ATP5F1 were significantly reduced after WDR12 knockdown, while the total mTOR protein levels did not show significant changes (Figure S1). These findings suggest that WDR12 plays a crucial role in regulating cancer progression-related pathways.

### Association between WDR12 expression and tumor microenvironments

**Fig. 5:**
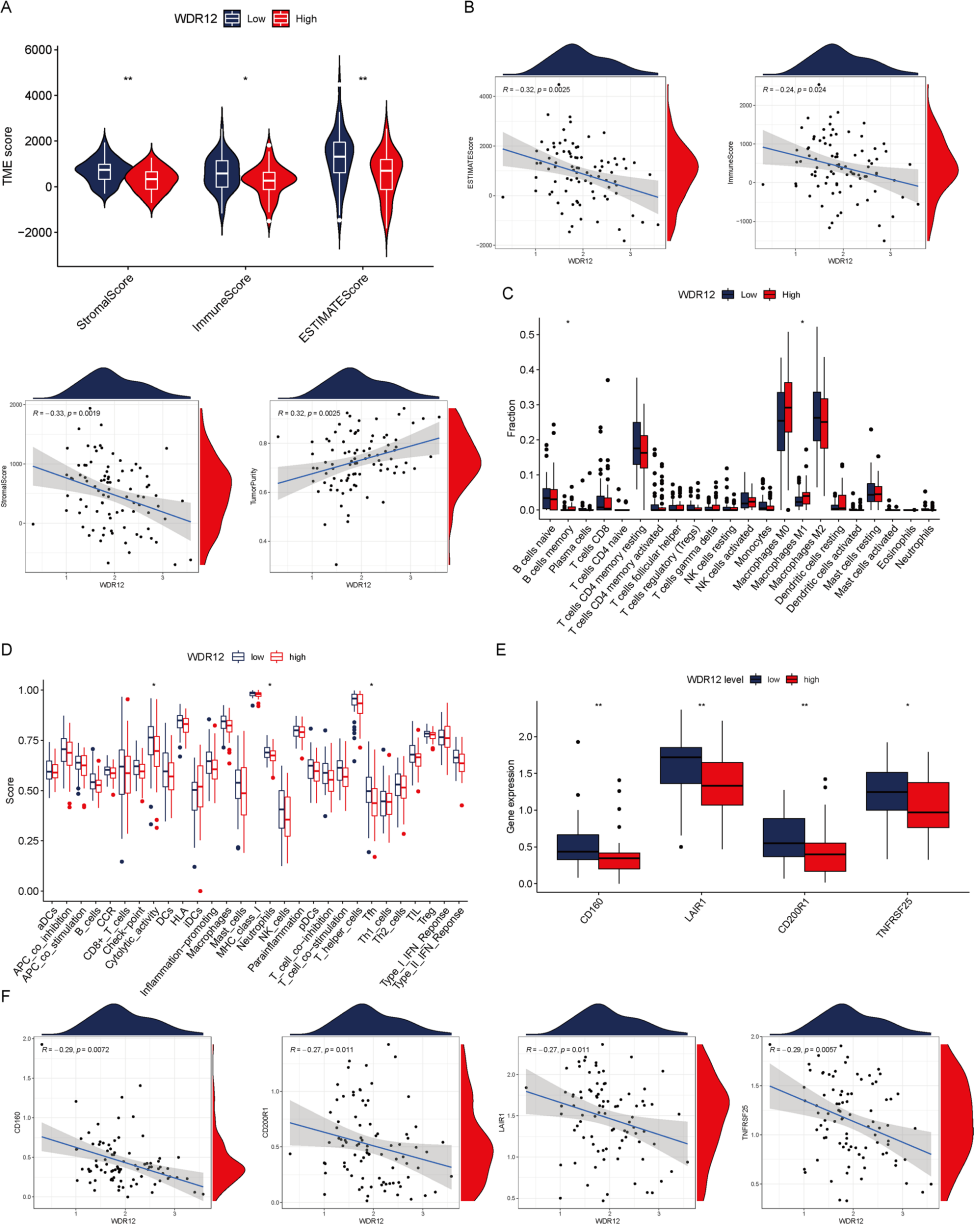
Immune infiltration pattern analysis of WDR12 in OS. (**A**) The difference in stromal score, immune score, and estimate score between the low and high WDR12 expression group. (**B**) The correlation between WDR12 and estimate score, immune score, stromal score, and estimate score. (**C**) CIBERSORT algorithm analyzes the distribution of the abundance of immune cell infiltration between the low and high WDR12 expression group. (**D**) The ssGSEA indicated the difference in enrichment scores of immune cells and immune functions between the low and high WDR12 expression group. (**E**) Differential expression of immune checkpoint genes in different WDR12 expression groups. (**F**) Relevance of expression between WDR12 and immune checkpoint genes

To explore the relationship between WDR12 expression and the tumor microenvironment in OS, the ESTIMATE algorithm was applied to calculate stromal, immune, and ESTIMATE scores. As shown in Fig. 5A, tumors in the low WDR12 expression group exhibited significantly higher stromal, immune, and ESTIMATE scores compared with those in the high WDR12 group. Correlation analyses further demonstrated that WDR12 expression was negatively associated with stromal, immune, and ESTIMATE scores, while positively correlated with tumor purity (Fig. 5B).

Immune cell infiltration was subsequently evaluated using the CIBERSORT algorithm. The results indicated that tumors with high WDR12 expressions were associated with higher proportions of memory B cells and M1 macrophages compared with the low WDR12 group (Fig. 5C). Correlation analysis confirmed a positive association between WDR12 expression and M1 macrophage infiltration (Figure S2). In addition, WDR12 expressions showed a positive correlation with STAT1, a key transcription factor involved in macrophage activation (Figure S2). These findings suggest a potential association between WDR12 expression and macrophage-related immune features, although a direct regulatory role requires further experimental validation. Consistent with these observations, ssGSEA analysis revealed that cytolytic activity, neutrophils, and T follicular helper (Tfh) cell signatures were relatively enriched in the low WDR12 expression group (Fig. 5D). Furthermore, the expression levels of several immune checkpoint–related molecules, including CD160, LAIR1, CD200R1, and TNFRSF25, were significantly higher in the low WDR12 group (Fig. 5E), and correlation analyses indicated significant associations between WDR12 expression and these immune checkpoint genes (Fig. 5F). Collectively, these results indicate that WDR12 expression is closely associated with variations in the immune landscape of OS.

### Prediction of therapeutic response and drug sensitivity

**Fig. 6:**
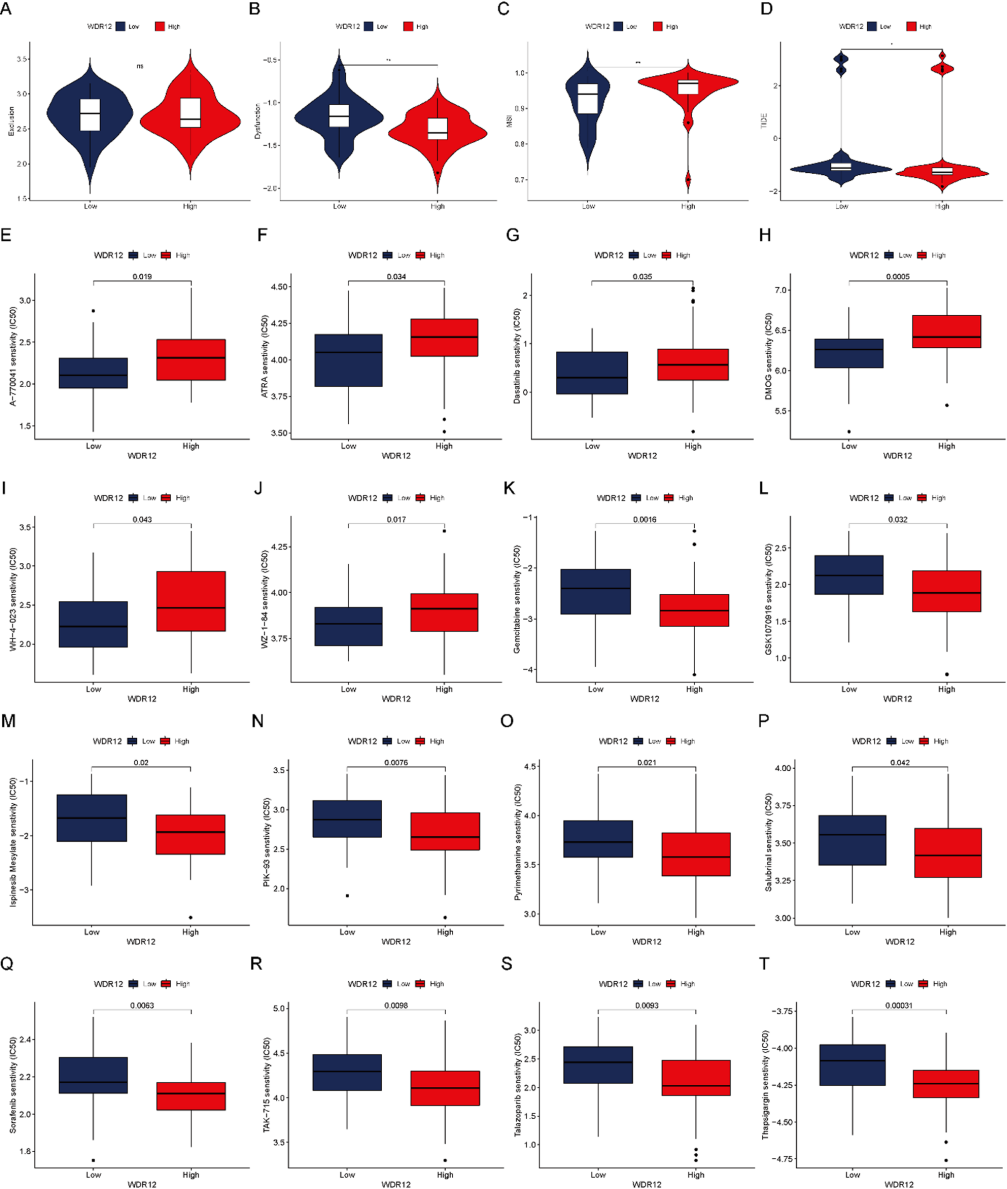
Prediction of treatment responses of OS patients. (**A**) The difference in Exclusion score between the low and high WDR12 expression group cohort. (**B**) The difference in Dysfunction score between the low and high WDR12 expression group cohort. (**C**) The difference in MSI score between the low and high WDR12 expression group cohort. (**D**) The difference in TIDE score between the low and high WDR12 expression group cohort. (**E-T**) The IC50 values of A-770,041, ATRA, Dasatinib, DMOG, WH-4–023, WZ-1–84, Gemcitabine, GSK1070916, Ispinesib, PIK-93, Pyrimethamine, Salubrinal, Sorafenib, TAK-715, Talazoparib, and Thapsigargin in different WDR12 expression groups

Given the observed associations between WDR12 expression and immune-related features, we further explored whether WDR12 expression was correlated with predicted responses to immunotherapy and chemotherapy. Analysis of immunotherapy-related indicators showed that the low WDR12 expression group exhibited higher TIDE and dysfunction scores, whereas the MSI score was relatively higher in the high WDR12 group (Fig. 6A–D), suggesting potential differences in immunotherapy response between the two groups. In addition, drug sensitivity analysis revealed that patients with low WDR12 expression had lower predicted IC50 values for A-770,041, ATRA, Dasatinib, DMOG, WH-4–023, and WZ-1–84 (all *P* < 0.05, Fig. 6E–J). Conversely, patients with high WDR12 expression showed lower predicted IC50 values for Gemcitabine, GSK1070916, Ispinesib, PIK-93, Pyrimethamine, Salubrinal, Sorafenib, TAK-715, Talazoparib, and Thapsigargin (all *P* < 0.05, Fig. 6K–T). These findings indicate that WDR12 expression is associated with differential drug sensitivity patterns in OS, although experimental validation is required to confirm these predictions.

### Single cell analysis

**Fig. 7:**
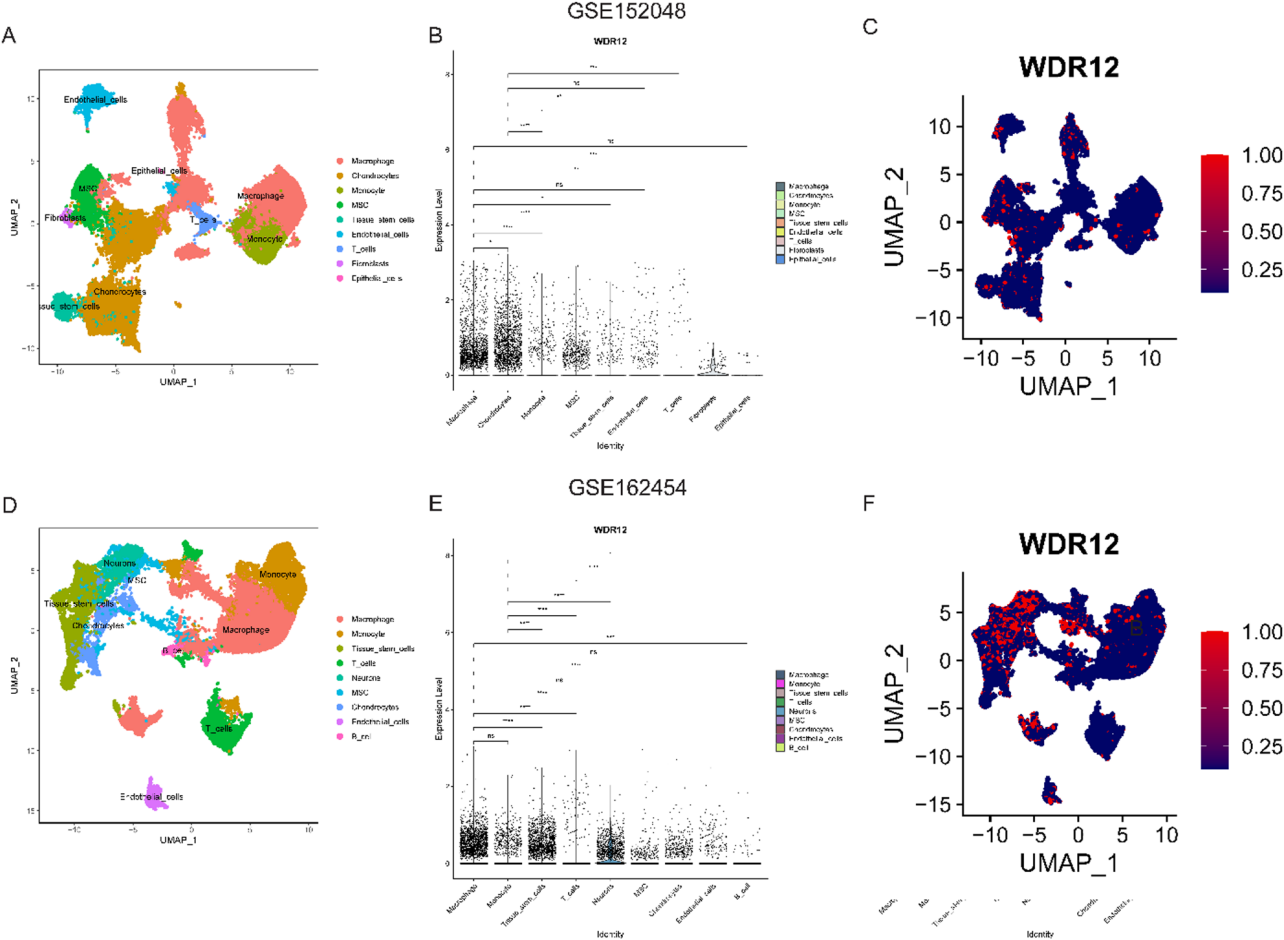
The single-cell landscape of WDR12 in OS. (**A**) Cell types were identified based on marker genes from the GSE152048 dataset. (**B-C**) WDR12 expression in various cells based on the GSE152048 dataset. (**D**) Cell types were identified based on marker genes from the GSE162454 dataset. (**E-F**) WDR12 expression in various cells based on the GSE162454 dataset

The expression of WDR12 in diverse cell types was examined in this study using two OS single-cell datasets (GSE152048 and GSE162454). The results revealed variations in the expression of WDR12 across different cells. Figure 7A and D provide an overview of the cell distribution in the GSE152048 and GSE162454 datasets, respectively. Figure 7B depicts the distribution of WDR12 in each cell within the GSE152048 dataset. Notably, WDR12 is observed in epithelial cells, MSCs, and tissue stem cells in this dataset, as displayed in Fig. 7C. Figure 7E demonstrates the distribution of WDR12 in various cell types in the GSE162454 dataset. In this dataset, WDR12 exhibits different expression levels in macrophages, monocytes, tissue stem cells, T cells, neurons, MSCs, chondrocytes, endothelial cells, and B cells. Particularly, neurons, tissue stem cells, and macrophages exhibit a higher concentration of WDR12, as shown in Fig. 7F. These findings highlight the multifaceted role of WDR12 and provide a foundation for future studies to explore its precise contributions to tumor behavior.

### The function of WDR12 in OS

**Fig. 8:**
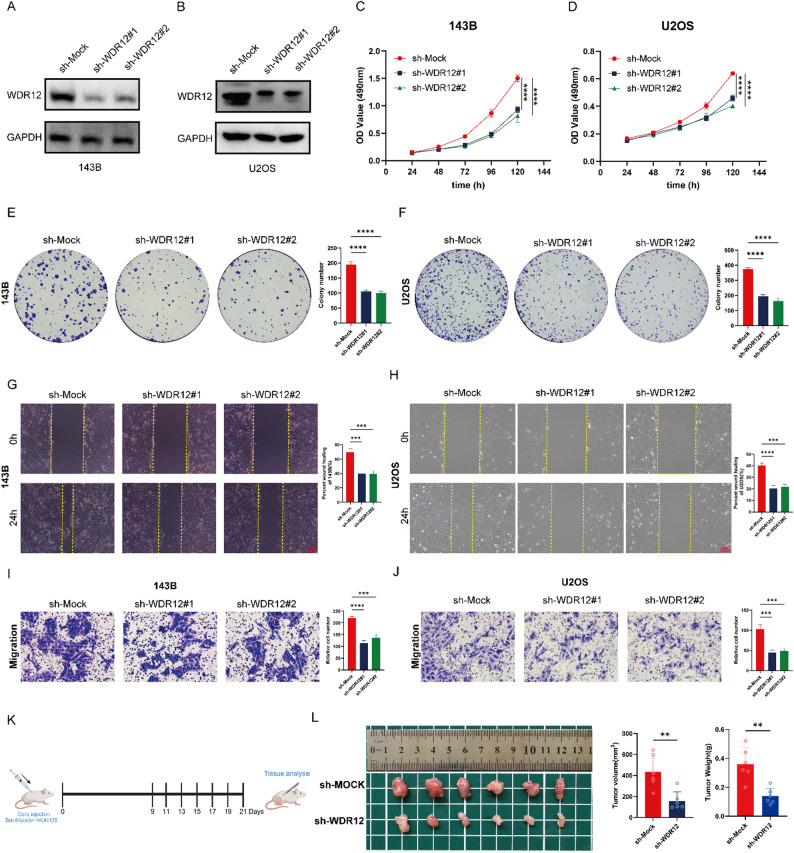
Impact of WDR12 knockdown on proliferation and migration ability of OS cells. (**A-B**) The knockdown efficiency of shRNA-WDR12 in 143B and U2OS was detected by WB. (**C-D**) MTT assay to evaluate changes in proliferation capacity of 143B and U2OS cells after WDR12 knockdown. (**E-F**) Colony formation assay to assess differences in proliferation capacity of 143B and U2OS cells after WDR12 knockdown. (**G-H**) Wound healing assay to assess changes in migration capacity of 143B and U2OS cells after WDR12 knockdown. (**I-J**) Transwell assay to evaluate changes in migration and invasion capacity of 143B and U2OS cells after WDR12 knockdown. (**K**) The flaw chart of animal experiments. (**L**) Tumor size and weight three weeks post-transplantation

To validate the reliability of our analysis, we conducted in vitro experiments to explore the role of WDR12 in OS cells. Initially, we utilized shRNA to suppress the expression of WDR12 in OS cell lines (143B, U2OS and HOS). As observed in Fig. 8A-B and Figure S3, the expression levels of WDR12 in 143B, U2OS and HOS cells were significantly downregulated in the sh-WDR12#1 and sh-WDR12#2 groups compared to the sh-Mock group. Subsequently, we performed MTT assays to evaluate the impact of altered WDR12 expression on the proliferative capacity of 143B, U2OS and HOS cells. The results demonstrated that the proliferation rates of 143B, U2OS and HOS cells in the sh-WDR12#1 and sh-WDR12#2 groups were markedly lower than those in the normal control group (Fig. 8C-D and Figure S3). Colony formation assays also indicated that the average number of colonies in the sh-Mock group of 143B, U2OS, and HOS cells was significantly higher than that in the sh-WDR12#1 and sh-WDR12#2 groups (Fig. 8E-F and Figure S3). Furthermore, we conducted wound healing and Transwell assays. Wound healing assays showed that the average wound healing rate after 24 h of the sh-Mock group in 143B, U2OS, and HOS cell lines was significantly higher than that of the sh-WDR12#1 and sh-WDR12#2 groups (Fig. 8G-H and Figure S3). Additionally, Transwell analysis showed that the average number of cells migrating through the chamber in the sh-Mock group of 143B, U2OS, and HOS cell lines was significantly higher than that in the sh-WDR12#1 and sh-WDR12#2 groups (Fig. 8I-J and Figure S3). Lastly, we conducted in vivo experiments to validate the effect of WDR12 on OS proliferation (Fig. 8K). As shown in Fig. 8L, WDR12 knockout significantly reduced the size and weight of tumors in nude mice compared to the control group. Consequently, these findings indicate that WDR12 enhances the proliferative and migratory capabilities of OS cells, consequently contributing to an unfavorable prognosis in OS.

## Discussion

OS is a malignant primary bone cancer predominantly affecting the pediatric and young adult population [21]. Surgical resection, in conjunction with chemotherapy, stands as the current cornerstone of OS treatment [22]. However, the disease’s high malignancy and intricate pathogenesis often lead to suboptimal treatment outcomes, highlighting the pressing necessity for an in-depth understanding of the molecular mechanisms dictating OS progression to inform the development of innovative therapeutic modalities. WD repeat sequences, characterized by around 40 amino acids and usually flanked by tryptophan-aspartate and glycine-histidine residues, serve as the smallest conserved regions capable of fostering the assembly of heterotrimeric or multiprotein complexes [23]. Within the WD repeats family, WDR12 exerts regulatory control over a spectrum of physiological processes, encompassing muscle differentiation, cardiac growth, T-regulatory cell function, and neurological conditions [24, 25]. Recent investigations have underscored the pivotal role of WDR12 in tumorigenesis, demonstrating its dual functionality across various cancer types [9]. Nevertheless, the specific involvement of WDR12 in OS pathogenesis remains obscure.

The utilization of public databases such as TARGET, TCGA, and GEO has played a crucial role in advancing our understanding of gene backgrounds, driver mutations, and essential pathways involved across various cancer types. In this study, we initially characterized abnormal expression patterns of WDR12 in different tumor types. Subsequently, by harnessing data from the TARGET and GEO repositories, we identified heightened levels of WDR12 expression in OS tissues, a finding corroborated through transcriptome sequencing and RT-qPCR analyses. Moreover, our research unveiled a significant association between WDR12 expression and the prognosis of OS patients, indicating its potential as an adverse prognostic marker for OS through survival and COX regression analyses. To refine prognostic evaluation in OS patients, we devised a nomogram integrating WDR12 expression and clinical attributes of OS cases, providing valuable insights for future prognostic assessments in OS cases.

Subsequently, we conducted co-expression and functional enrichment analyses to elucidate potential genes and signaling pathways linked to WDR12. Remarkably, our investigation revealed a significant enrichment of cancer-related pathways in OS patients exhibiting elevated WDR12 expression, including DNA repair, MTORC1 signaling, MYC targets V1, MYC targets V2, and oxidative phosphorylation, among others. These pathways are known to exert crucial roles in the initiation and progression of tumors. For instance, cysteine within the nutrient composition has been shown to enhance colon cancer growth and resistance to chemotherapy through mTORC1 activation and reactive oxygen species (ROS) clearance [26]. MYC, as a transcription factor, plays a pivotal role in key biological functions such as cell proliferation, differentiation, and apoptosis, serving as a significant driver in various cancer types like breast, lung, and colon cancers [27–29]. Oxidative phosphorylation, a fundamental cellular process occurring in mitochondria, generates energy through redox reactions to produce adenosine triphosphate (ATP) for cell sustenance [30]. The role of oxidative phosphorylation in different cancers remains somewhat variable. Research by Yao ZK et al. has demonstrated that Manoalide induces endogenous apoptosis in human OS cells via oxidative stress and mitochondrial dysfunction [31]. These events potentially contribute to the unfavorable prognosis observed in OS patients with heightened WDR12 expression.

This study reveals the expression of WDR12 not only in malignant cells but also in immune cells and stromal cells, implying a significant association between WDR12 and the tumor immune microenvironment, potentially mediated through diverse cellular interactions. Comparative analysis showed that in the low WDR12 expression group, stromal cell and immune cell scores were higher, while tumor scores were lower. Immune cell profiling demonstrated a positive correlation between reduced WDR12 expression and increased infiltration of specific immune cells, such as neutrophils. Neutrophils can target and eliminate tumor cells, impeding their growth, migration, and invasion, thereby enhancing cancer patient prognosis [32]. Our findings indicated a higher neutrophil presence in OS patients with low WDR12 expression compared to those with high WDR12 expression, possibly contributing to the unfavorable prognosis observed in the latter group. M1 macrophages, known for their polarized nature, play a dual role in cancer progression [33]. In early-stage lung cancer, M1 macrophages can eliminate tumor cells and stimulate T cell-mediated immune responses for enhanced tumor eradication [34, 35]. Conversely, in advanced lung cancer stages, M1 macrophages may promote tumor proliferation and metastasis [36]. Interestingly, our investigation revealed a higher abundance of M1 macrophages in patients with high WDR12 expression, suggesting their potential involvement in the growth and spread of OS cells. Furthermore, we observed a significant positive correlation between WDR12 and STAT1. As is well-established, STAT1 plays a crucial role in the polarization and function of M1-type macrophages [37]. Therefore, we hypothesize that WDR12 may influence M1 macrophage polarization by regulating STAT1 expression, thereby affecting the survival outcomes of OS patients. This insight holds significant potential for understanding the differences in the immune microenvironment of OS and guiding therapeutic strategies.

Furthermore, our study revealed notable variations in the expression of diverse immune checkpoint genes across distinct WDR12 expression groups, implying a potential role of WDR12 expression levels in guiding the identification of future immunotherapy targets for OS. Likewise, differences were observed in Dysfunction, MSI, and TIDE scores among patients with varying WDR12 expression levels, suggesting the utility of WDR12 as a molecular biomarker for tailored immunotherapy in OS cases. These findings open opportunities for exploring immunotherapeutic strategies in OS. For instance, combining WDR12-targeted interventions with immune checkpoint inhibitors, such as anti-PD-1/PD-L1 therapies, could synergistically enhance anti-tumor immunity. Such combinatorial strategies may improve the efficacy of immunotherapy, particularly in OS patients with high WDR12 expression. Of course, future research, including preclinical studies and clinical trials, is necessary to explore the translational potential of these findings. Subsequent analysis of treatment responses included an evaluation of variation in IC50 values for various chemotherapy agents across cohorts with differing WDR12 expression levels. The findings indicate that patients displaying low WDR12 expression exhibit enhanced responsiveness to drugs such as Dasatinib in comparison to those with high WDR12 expression. This phenomenon is postulated to be associated with elevated gene mutation frequencies, heightened malignancy, and increased proliferation rates in these individuals. Considering this observation, the development of drugs specifically targeting WDR12, such as selective inhibitors, PROTACs, or degraders, holds significant potential for improving the prognosis of OS patients. Furthermore, tailoring drug selection based on WDR12 expression levels could enhance treatment efficacy, underscoring the critical role of WDR12 in advancing personalized precision therapy.

To enhance the validity of our study, we conducted in vitro experiments to ascertain the impact of WDR12 on the malignant biological behavior of OS. The MTT, colony formation, scratch healing, transwell assays and in vivo experiment results revealed that the downregulation of WDR12 significantly impeded the proliferation and migration capabilities of OS cells. These outcomes align with existing literature. For instance, Su Wen et al. demonstrated that WDR12 promotes the proliferation and suppresses apoptosis of colorectal cells by regulating RAC1 expression, and its knockdown inhibits colorectal cell proliferation [38]. Additionally, Jun-Liang Li illustrated that in glioma, WDR12 is upregulated, and its suppression induces apoptosis while impeding proliferation in glioma cells [9]. By integrating our results with these prior studies, we present compelling evidence to indicate the potential of WDR12 as a prospective biomarker for enhancing the prognosis of OS.

Notwithstanding the significance of our findings, several limitations necessitate acknowledgement. First, our analyses relied predominantly on retrospective public datasets (TARGET, TCGA, and GEO), which inherently carry unavoidable selection biases. Furthermore, the histological heterogeneity of the TCGA-SARC cohort, coupled with incomplete clinical annotations, precluded strict stratification of OS-specific subgroups, potentially limiting the interpretability of our results. Validation in larger, well-annotated clinical cohorts is thus essential. Second, while we conducted in vitro functional assays across multiple cell lines, our mechanistic insights remain preliminary. Although we validated representative downstream genes involved in key oncogenic pathways following WDR12 knockdown, the precise signaling cascades governed by WDR12 have yet to be fully delineated. Notably, rescue experiments were not performed to definitively establish a direct causal relationship between WDR12 depletion and the observed phenotypic changes. Third, associations between WDR12 expression and drug sensitivity were predicted solely via bioinformatic approaches and lack experimental verification. Future investigations must prioritize the systematic evaluation of therapeutic responses using complementary in vitro and vivo systems. Finally, our immune infiltration analyses relied on computational estimation and correlative statistics; thus, conclusions are limited to associations rather than direct regulatory mechanisms. Further functional validation is required to clarify whether WDR12 directly modulates the tumor immune microenvironment.

## Conclusion

In summary, our study reveals that elevated WDR12 expression is correlated with unfavorable survival outcomes and aggressive malignant phenotypes in OS. WDR12 expression is associated with immune status and therapeutic response profiles, suggesting that it may represent a promising prognostic biomarker and candidate therapeutic target for OS, pending further mechanistic and clinical validation.

## Supplementary Information

Supplementary Material 1

Supplementary Material 2

Supplementary Material 3

Supplementary Material 4

Supplementary Material 5

## Data Availability

Data sources and handling of the publicly available data sets used in our study are described in the Materials and Methods; all RNA-seq and clinical data sets are available on TARGET (https://ocg.cancer.gov/programs/target), GEO (https://www.ncbi.nlm.nih.gov/geo/) and TCGA (https://portal.gdc.cancer.gov/) database. R code and all other data used in the manuscript can be provided when necessary.

## Abbreviations

OS: Osteosarcoma
TARGET: Therapeutically Applicable Research to Generate Effective Treatments database
GEO: Gene Expression Omnibus
TCGA: The Cancer Genome Atlas
DCA: Decision curve analysis
GO: Gene Ontology
KEGG: Kyoto Encyclopedia of Genes and Genomes
GSEA: Gene set enrichment analysis
ssGSEA: Single-sample Gene set enrichment analysis
TIDE: Tumor Immune Dysfunction and Exclusion
MSI: Microsatellite instability
GDSC: Genomics of Drug Sensitivity in Cancer
IC50: Half-maximal inhibitory concentration
ATCC: American Type Culture Collection

## References

[1] Rav E, Maegawa S, Gopalakrishnan V, Gordon N. Overview of CD70 as a potential therapeutic target for osteosarcoma. J Immunol. 2023;211(7):1067–72. 37722095 10.4049/jimmunol.2200591

[2] Li Y, Nakka M, Kelly AJ, et al. p27 Is a Candidate Prognostic Biomarker and Metastatic Promoter in Osteosarcoma. Cancer Res. 2016;76(13):4002–11. 27197201 10.1158/0008-5472.CAN-15-3189 PMC4930684

[3] Tian H, Cao J, Li B, et al. Managing the immune microenvironment of osteosarcoma: the outlook for osteosarcoma treatment. Bone Res. 2023;11(1):11. 36849442 10.1038/s41413-023-00246-z PMC9971189

[4] Lu KH, Lu PW, Lu EW, Lin CW, Yang SF. Curcumin and its analogs and carriers: potential therapeutic strategies for human osteosarcoma. Int J Biol Sci. 2023;19(4):1241–65. 36923933 10.7150/ijbs.80590 PMC10008701

[5] Beird HC, Bielack SS, Flanagan AM, et al. Osteosarcoma. Nat Rev Dis Primers. 2022;8(1):77. 36481668 10.1038/s41572-022-00409-y

[6] Zhao J, Dean DC, Hornicek FJ, Yu X, Duan Z. Emerging next-generation sequencing-based discoveries for targeted osteosarcoma therapy. Cancer Lett. 2020;474:158–67. 31987920 10.1016/j.canlet.2020.01.020

[7] Hölzel M, Rohrmoser M, Schlee M, et al. Mammalian WDR12 is a novel member of the Pes1-Bop1 complex and is required for ribosome biogenesis and cell proliferation. J Cell Biol. 2005;170(3):367–78. 16043514 10.1083/jcb.200501141 PMC2171466

[8] Khoshnevis S, Askenasy I, Johnson MC, et al. The DEAD-box protein Rok1 orchestrates 40S and 60S ribosome assembly by promoting the release of Rrp5 from pre-40S ribosomes to allow for 60S maturation. PLoS Biol. 2016;14(6):e1002480. 27280440 10.1371/journal.pbio.1002480 PMC4900678

[9] Li JL, Chen C, Chen W, et al. Integrative genomic analyses identify WDR12 as a novel oncogene involved in glioblastoma. J Cell Physiol. 2020;235(10):7344–55. 32180229 10.1002/jcp.29635

[10] Yin Y, Zhou L, Zhan R, Zhang Q, Li M. Identification of WDR12 as a novel oncogene involved in hepatocellular carcinoma propagation. Cancer Manag Res. 2018;10:3985–93. 30310320 10.2147/CMAR.S176268 PMC6166768

[11] Trang NTN, Lai CY, Tsai HC, et al. Apelin promotes osteosarcoma metastasis by upregulating PLOD2 expression via the Hippo signaling pathway and hsa_circ_0000004/miR-1303 axis. Int J Biol Sci. 2023;19(2):412–25. 36632453 10.7150/ijbs.77688 PMC9830518

[12] Chen W, Lin Y, Jiang M, Wang Q, Shu Q. Identification of LARS as an essential gene for osteosarcoma proliferation through large-Scale CRISPR-Cas9 screening database and experimental verification. J Transl Med. 2022;20(1):355. 35962451 10.1186/s12967-022-03571-9 PMC9373537

[13] Shen H, Wang W, Ni B, Zou Q, Lu H, Wang Z. Exploring the molecular mechanisms of osteosarcoma by the integrated analysis of mRNAs and miRNA microarrays. Int J Mol Med. 2018;42(1):21–30. 29620143 10.3892/ijmm.2018.3594 PMC5979835

[14] Chen W, Liao Y, Sun P, et al. Construction of an ER stress-related prognostic signature for predicting prognosis and screening the effective anti-tumor drug in osteosarcoma. J Transl Med. 2024;22(1):66. 38229155 10.1186/s12967-023-04794-0 PMC10792867

[15] Kanehisa M, Furumichi M, Sato Y, Matsuura Y, Ishiguro-Watanabe M. KEGG: biological systems database as a model of the real world. Nucleic Acids Res. 2025;53(D1):D672–7. 39417505 10.1093/nar/gkae909 PMC11701520

[16] Zhou Y, Yang D, Yang Q, et al. Single-cell RNA landscape of intratumoral heterogeneity and immunosuppressive microenvironment in advanced osteosarcoma. Nat Commun. 2020;11(1):6322. 33303760 10.1038/s41467-020-20059-6 PMC7730477

[17] Liu Y, Feng W, Dai Y, et al. Single-cell transcriptomics reveals the complexity of the tumor microenvironment of treatment-naive osteosarcoma. Front Oncol. 2021;11:709210. 34367994 10.3389/fonc.2021.709210 PMC8335545

[18] Huang X, Wang L, Guo H, Zhang W, Shao Z. Single-cell transcriptomics reveals the regulative roles of cancer associated fibroblasts in tumor immune microenvironment of recurrent osteosarcoma. Theranostics. 2022;12(13):5877–87. 35966586 10.7150/thno.73714 PMC9373820

[19] Liu Z, Liu B, Feng C, et al. Molecular characterization of immunogenic cell death indicates prognosis and tumor microenvironment infiltration in osteosarcoma. Front Immunol. 2022;13:1071636. 36569869 10.3389/fimmu.2022.1071636 PMC9780438

[20] Tu C, Liu B, Li C et al. Integrative analysis of TROAP with molecular features, carcinogenesis, and related immune and pharmacogenomic characteristics in soft tissue sarcoma. MedComm (2020). 2023;4(5):e369. 10.1002/mco2.369 PMC10507284 37731946

[21] Mirabello L, Troisi RJ, Savage SA. International osteosarcoma incidence patterns in children and adolescents, middle ages and elderly persons. Int J Cancer. 2009;125(1):229–34. 19330840 10.1002/ijc.24320 PMC3048853

[22] Meltzer PS, Helman LJ. New horizons in the treatment of osteosarcoma. N Engl J Med. 2021;385(22):2066–76. 34818481 10.1056/NEJMra2103423

[23] Raab M, Daxecker H, Edwards RJ, Treumann A, Murphy D, Moran N. Protein interactions with the platelet integrin alpha(IIb) regulatory motif. Proteomics. 2010;10(15):2790–800. 20486118 10.1002/pmic.200900621

[24] Yuan Y, Qi G, Shen H, et al. Clinical significance and biological function of WD repeat domain 54 as an oncogene in colorectal cancer. Int J Cancer. 2019;144(7):1584–95. 29987896 10.1002/ijc.31736

[25] Xu W, Grain D, Bobet S, et al. Complexity and robustness of the flavonoid transcriptional regulatory network revealed by comprehensive analyses of MYB-bHLH-WDR complexes and their targets in Arabidopsis seed. New Phytol. 2014;202(1):132–44. 24299194 10.1111/nph.12620

[26] Wu J, Yeung SJ, Liu S. Cyst(e)ine in nutrition formulation promotes colon cancer growth and chemoresistance by activating mTORC1 and scavenging ROS. Signal Transduct Target Ther. 2021;6(1):188. 34045438 10.1038/s41392-021-00581-9 PMC8160199

[27] Einstein JM, Perelis M, Chaim IA, et al. Inhibition of YTHDF2 triggers proteotoxic cell death in MYC-driven breast cancer. Mol Cell. 2021;81(15):3048-64.e9. 34216543 10.1016/j.molcel.2021.06.014 PMC8359670

[28] Ireland AS, Micinski AM, Kastner DW, et al. MYC drives temporal evolution of small cell lung cancer subtypes by reprogramming neuroendocrine fate. Cancer Cell. 2020;38(1):60-78.e12. 32473656 10.1016/j.ccell.2020.05.001 PMC7393942

[29] Saeed H, Leibowitz BJ, Zhang L, Yu J. Targeting Myc-driven stress addiction in colorectal cancer. Drug Resist Updat. 2023;69:100963. 37119690 10.1016/j.drup.2023.100963 PMC10330748

[30] Ashton TM, McKenna WG, Kunz-Schughart LA, Higgins GS. Oxidative phosphorylation as an emerging target in cancer therapy. Clin Cancer Res. 2018;24(11):2482–90. 29420223 10.1158/1078-0432.CCR-17-3070

[31] Yao ZK, Jean YH, Lin SC, et al. Manoalide Induces Intrinsic Apoptosis by Oxidative Stress and Mitochondrial Dysfunction in Human Osteosarcoma Cells. Antioxidants. 2023. 10.3390/antiox12071422. 37507960 10.3390/antiox12071422 PMC10376204

[32] Ley K, Hoffman HM, Kubes P, et al. Neutrophils: New insights and open questions. Sci Immunol. 2018. 10.1126/sciimmunol.aat4579. 30530726 10.1126/sciimmunol.aat4579

[33] Mantovani A, Marchesi F, Malesci A, Laghi L, Allavena P. Tumour-associated macrophages as treatment targets in oncology. Nat Rev Clin Oncol. 2017;14(7):399–416. 28117416 10.1038/nrclinonc.2016.217 PMC5480600

[34] Litchfield K, Reading JL, Puttick C, et al. Meta-analysis of tumor- and T cell-intrinsic mechanisms of sensitization to checkpoint inhibition. Cell. 2021;184(3):596-614.e14. 33508232 10.1016/j.cell.2021.01.002 PMC7933824

[35] Lee N, Heo YJ, Choi SE, et al. Anti-inflammatory effects of empagliflozin and gemigliptin on LPS-stimulated macrophage via the IKK/NF-κB, MKK7/JNK, and JAK2/STAT1 signalling pathways. J Immunol Res. 2021;2021:9944880. 34124273 10.1155/2021/9944880 PMC8192181

[36] Jackute J, Zemaitis M, Pranys D, et al. Distribution of M1 and M2 macrophages in tumor islets and stroma in relation to prognosis of non-small cell lung cancer. BMC Immunol. 2018;19(1):3. 29361917 10.1186/s12865-018-0241-4 PMC5781310

[37] Cao J, Ji L, Zhan Y, et al. MST4 kinase regulates immune thrombocytopenia by phosphorylating STAT1-mediated M1 polarization of macrophages. Cell Mol Immunol. 2023;20(12):1413–27. 37833401 10.1038/s41423-023-01089-8 PMC10687271

[38] Wen S, Huang X, Xiong L, et al. WDR12/RAC1 axis promoted proliferation and anti-apoptosis in colorectal cancer cells. Mol Cell Biochem. 2024;479(12):3341-54. 10.1007/s11010-024-04937-x. 38341833 10.1007/s11010-024-04937-x

