# Supplementary figures and images for: The prognostic value and oncogenic functions of WDR12 and its validation in osteosarcoma
Source: Cancer Cell Int. 2026 Apr 8;26:206. doi: 10.1186/s12935-026-04291-6 (PMC13214088; doi:10.1186/s12935-026-04291-6)

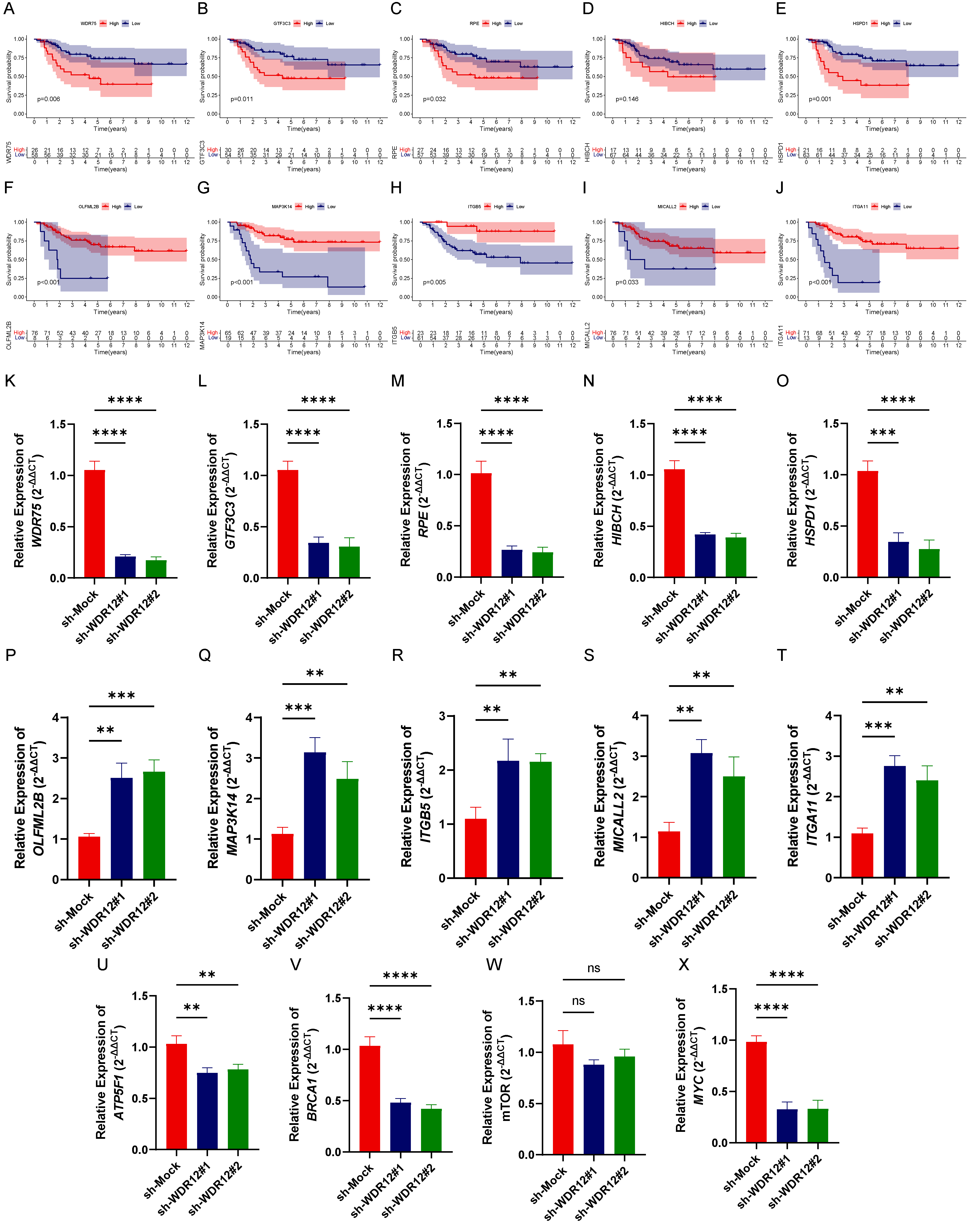

Supplement: Supplementary file 1 — Supplementary Material 1 [file 12935_2026_4291_MOESM1_ESM.tif]

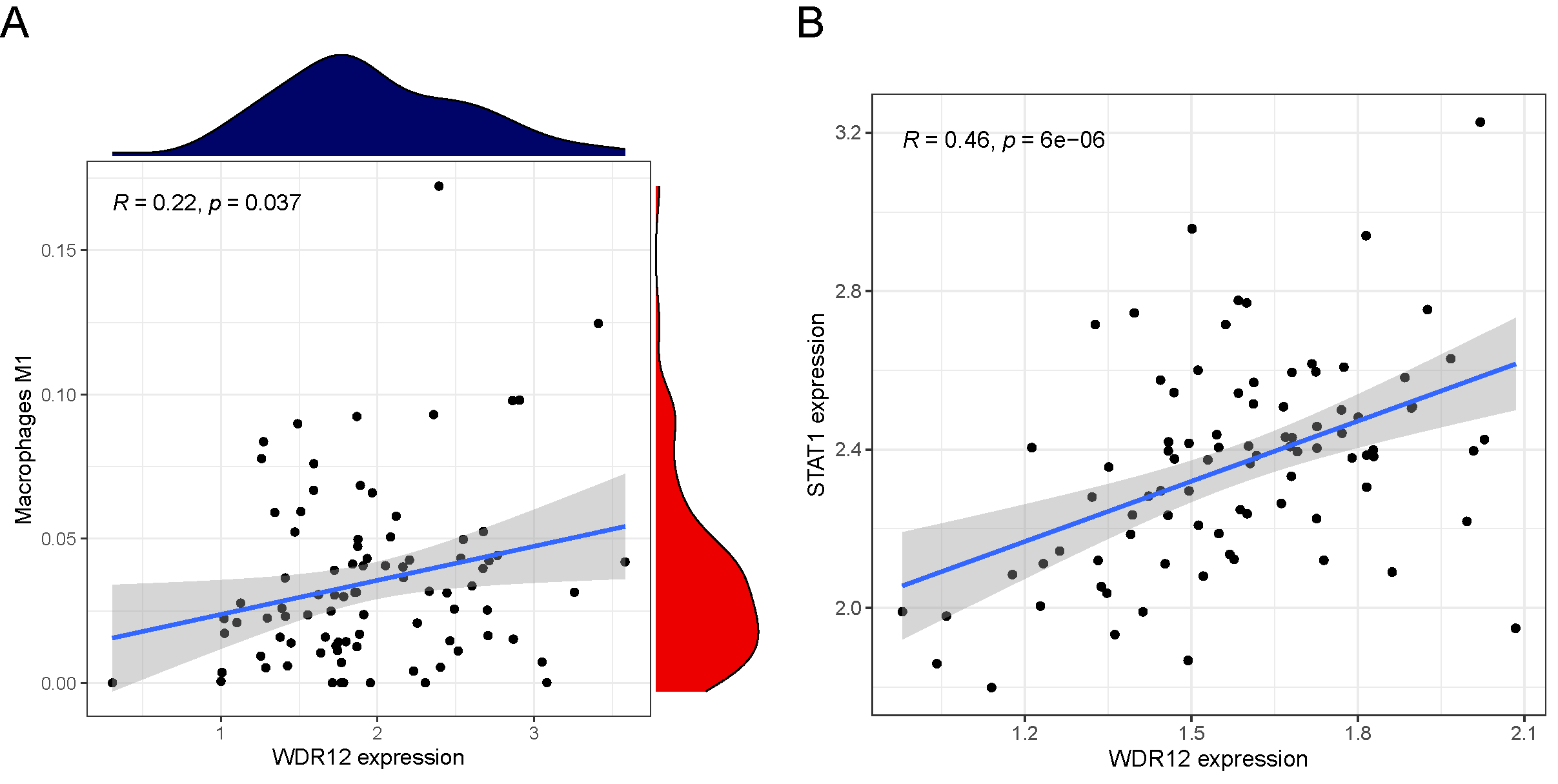

Supplement: Supplementary file 2 — Supplementary Material 2 [file 12935_2026_4291_MOESM2_ESM.tif]

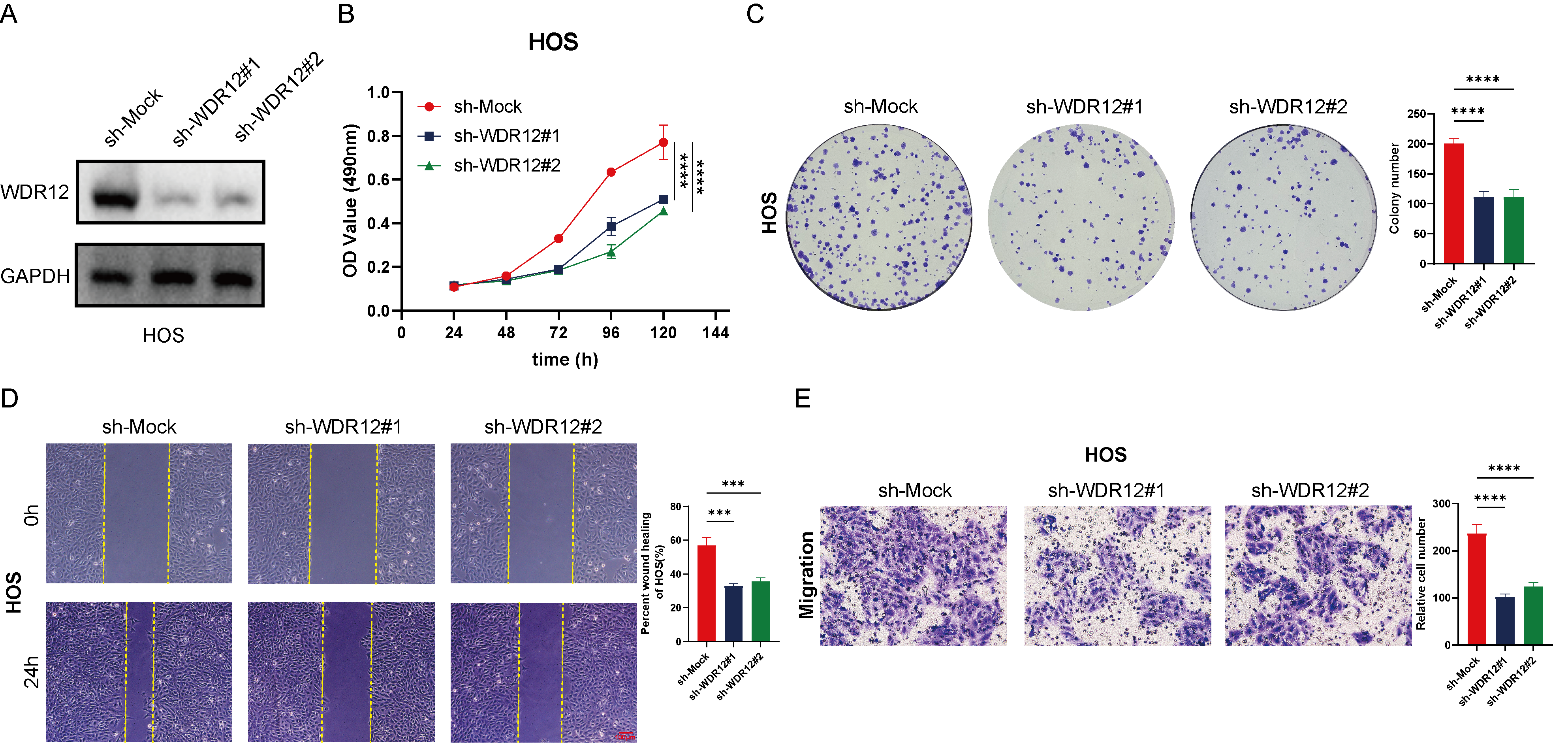

Supplement: Supplementary file 3 — Supplementary Material 3 [file 12935_2026_4291_MOESM3_ESM.tif]

Figure 1H

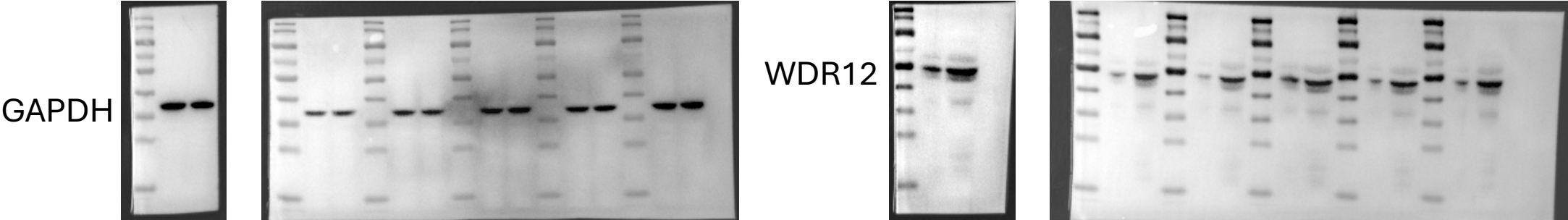

Figure 8A

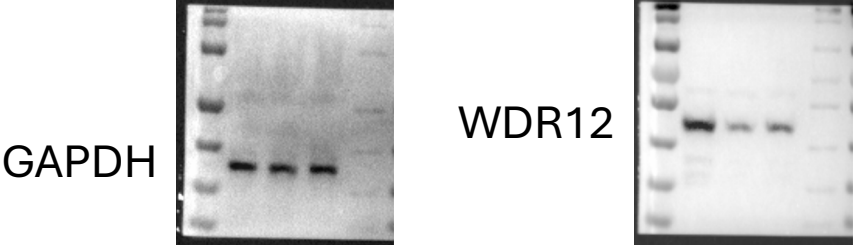

Figure 8B

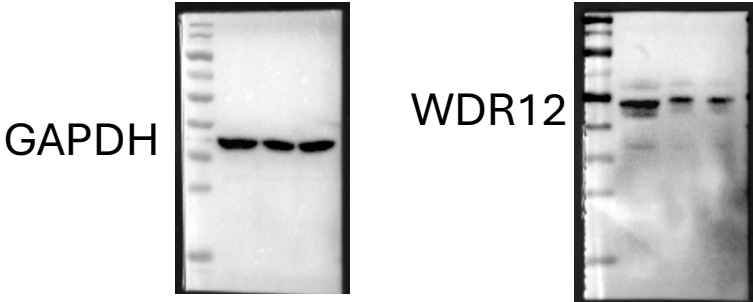

Figure S3A

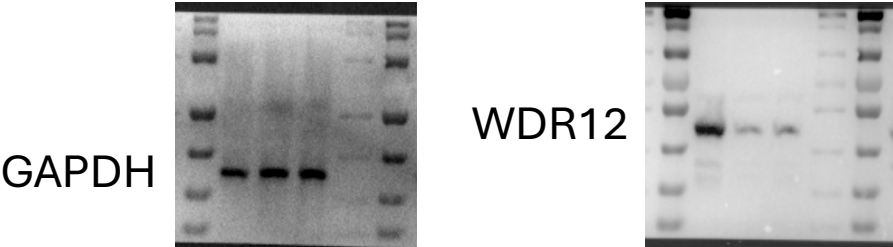

Supplement: Supplementary file 5 — Supplementary Material 5 [file 12935_2026_4291_MOESM5_ESM.pdf]
